# CT image-based 3D inflorescence estimation of *Chrysanthemum seticuspe*

**DOI:** 10.3389/fpls.2024.1374937

**Published:** 2024-07-29

**Authors:** Soushi Matsumoto, Yuzuko Utsumi, Toshiaki Kozuka, Masakazu Iwamura, Tomonori Nakai, Daisuke Yamauchi, Ichirou Karahara, Yoshinobu Mineyuki, Masato Hoshino, Kentaro Uesugi, Koichi Kise

**Affiliations:** ^1^ Graduate School of Informatics, Osaka Metropolitan University, Sakai, Japan; ^2^ Institute of Science and Engineering, Kanazawa University, Kanazawa, Japan; ^3^ Graduate School of Integrated Sciences for Life, Hiroshima University, Higashi-Hiroshima, Japan; ^4^ Graduate School of Science, University of Hyogo, Himeji, Japan; ^5^ School of Science, University of Toyama, Toyama, Japan; ^6^ Scattering and Imaging Division, Japan Synchrotron Radiation Research Institute, Sayo, Japan

**Keywords:** CT image, object detection, segmentation, clustering, 3D data analysis

## Abstract

To study plant organs, it is necessary to investigate the three-dimensional (3D) structures of plants. In recent years, non-destructive measurements through computed tomography (CT) have been used to understand the 3D structures of plants. In this study, we use the *Chrysanthemum seticuspe* capitulum inflorescence as an example and focus on contact points between the receptacles and florets within the 3D capitulum inflorescence bud structure to investigate the 3D arrangement of the florets on the receptacle. To determine the 3D order of the contact points, we constructed slice images from the CT volume data and detected the receptacles and florets in the image. However, because each CT sample comprises hundreds of slice images to be processed and each *C. seticuspe* capitulum inflorescence comprises several florets, manually detecting the receptacles and florets is labor-intensive. Therefore, we propose an automatic contact point detection method based on CT slice images using image recognition techniques. The proposed method improves the accuracy of contact point detection using prior knowledge that contact points exist only around the receptacle. In addition, the integration of the detection results enables the estimation of the 3D position of the contact points. According to the experimental results, we confirmed that the proposed method can detect contacts on slice images with high accuracy and estimate their 3D positions through clustering. Additionally, the sample-independent experiments showed that the proposed method achieved the same detection accuracy as sample-dependent experiments.

## 1 Introduction

Analysis of plant structures, such as mathematical modeling and classification of species based on morphological information, is necessary for botanical research. For example, in the study by Shaiju and Omanakumari (2009), the authors attempted to classify plants of the genus *Thottea* on the basis of quantitative and qualitative inflorescence and leaf characteristics. In a study by Yonekura et al. (2019), a mathematical model for generating phyllotaxis patterns was developed, and essential elements in the leaf formation process were identified by comparing the generated patterns with those of actual plants. Structural analysis of plants can typically be performed by visual observation.

Although common plant observations are destructive surveys, when the internal or three-dimensional (3D) structure of plants is analyzed, the sample is observed using non-destructive techniques (Pajor et al., 2013), such as computed tomography (CT). Micro-CT technique is a useful imaging tool that significantly contributes to the research of plant development by providing high-resolution, three-dimensional structural measurements (Karahara et al., 2023). This allows for non-destructive imaging to precisely quantify phenotypic traits and provide essential data for modeling plant growth and development, including the internal architecture. Also, high-throughput phenotyping evaluation in a large number of samples could be possible by establishing a segmentation pipeline that automatically and rapidly analyzes accurate plant structure using micro-CT data. This advancement has the potential to significantly contribute to the future development of crop breeding (Wu et al., 2021).

**Figure 1:**
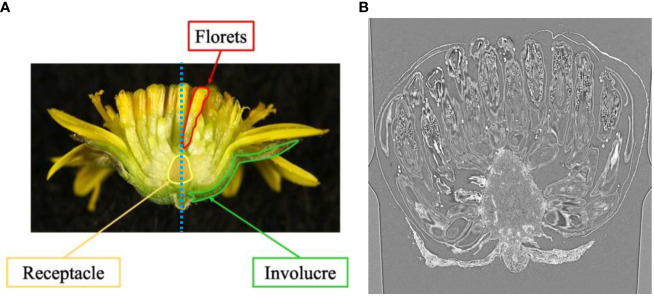
Example of mid-longitudinal section **(A)** and CT slice image of *C. seticuspe* **(B)**. The blue dotted line in **(A)** shows the rotation axis.

In this study, we use the capitulum inflorescence of *Chrysanthemum seticuspe* (Maxim.) Hand.-Mazz as an example and aim to estimate and analyze the 3D arrangement of the inflorescence. We chose *C. seticuspe* because its entire gene sequence has already been decoded and its transgenic plants can be generated (Nakano et al., 2021). **Figure 1A** shows the structure of the *C. seticuspe* capitulum inflorescence, which consists of a receptacle, florets, and involucre. To visualize the arrangement of the inflorescence, it is necessary to detect the contact points between the florets and receptacle. Because the bud size is small and the contact points are inside the bud, we captured CT volume data and analyzed the slice images shown in **Figure 1B** to estimate the 3D arrangement of the florets. By estimating the 3D positions of the contact points between the receptacle and the florets, we can analyze the floret arrangement pattern on the receptacle. Specifically, we calculate the 3D distance between adjacent florets and examine the relationship between this distance and their positions on the receptacle. This allows us to investigate how the receptacle’s meristem influences the 3D arrangement of the florets and will contribute to elucidating the mechanism of flower development in *C. seticuspe*.

In the previous study, Zhang et al. used micro-CT data and scanning electron microscope (SEM) images to estimate the inflorescence of gerbera florets, which belong to the same Asteraceae family as *C. seticuspe* (Zhang et al., 2021). In the previous study, CT cross-sections were used to observe the change in the shape of the receptacle with growth. The pattern of floret phyllotaxis was estimated from 2D SEM images taken from the top of the flower. However, neither segmentation of the receptacle in the CT cross-sections nor estimation of the 3D shape of the receptacle was performed. In addition, the 3D position of the contact points between the receptacle and the florets was not estimated. Therefore, it would be difficult for the previous method to perform the analysis using the distance between adjacent florets in 3D as planned in our study.

In addition, there are several studies that analyze plants using CT volume data. Most of them obtain the 3D shape of the plant and statistical information from the 3D shape. However, they cannot estimate the positional information of the 3D structure of the plant, such as the 3D arrangement of the contact points between the receptacle and the florets. For example (Tracy et al., 2017), succeeded in obtaining a 3D shape of Arabidopsis (*Arabidopsis thaliana* L.) and barley (*Hordeum vulgare* L.) flowers from CT volume data using simple image processing that distinguishes air and flower organs. Since the floral organs were not segmented on the obtained 3D shape, the information on the 3D positional relationship of each organ cannot be estimated (Mathers et al., 2018). used CT volume data of Arabidopsis, tomato, pea, barley, oat, and rice leaves to measure leaf porosity and mesophyll exposed surface area. This study extracted statistical data of leaf porosity and mesophyll exposed surface area from the volume data using simple image processing and did not analyze the 3D structure of leaf cells (Wu et al., 2021). takes images of the whole rice plant and performs semantic segmentation on 80 slices of the stem near the base to extract statistics related to the collapse of the rice plant, such as the area occupied by the stem. In addition (Teramoto et al., 2020), uses simple image processing to extract and visualize the 3D shape of roots from CT data, and extracts statistical information such as the total length of the roots. These are also statistical analyses based on the extracted shapes, not structural information as in our study. In (Ijiri et al., 2014), they propose a method to divide each petal of a flower into individual petals from the CT volume. This method is similar to our study because it can obtain 3D position information of the petals. However, it is difficult to apply this method because the shape of the flower in this study is different from that of the *C. seticuspe*, which has a large number of flowers on a single bud.

In this study, we propose an image processing method that uses *C. seticuspe* CT volume data to estimate the 3D position of the inflorescence. We obtained CT volume data of *C. seticuspe* bud, and then analyzed it using semantic and instance segmentation. To the best of our knowledge, this is the first attempt to apply instance and semantic segmentation to the CT volume data of *C. seticuspe* bud. Another novelty of this study is that the proposed method can obtain the 3D structural information, which cannot be obtained by the previous studies. We detected the point between the floret and receptacle using object detection on CT slice images. Because many regions are similar to the contact points on the CT slice images, the detection results contain false positives. Therefore, we detected the receptacles using semantic segmentation and excluded false positives not on the receptacles, thereby detecting contact points with high accuracy. The detected contact points are then integrated into 3D space. The contact points are not uniquely integrated in 3D space because the detection results contain positional errors and a contact point appears in multiple CT images. Therefore, a clustering algorithm is applied to the contact points in 3D space, and the average of each cluster is taken as the contact point estimation result.

We evaluated the proposed method on the basis of the original data obtained by the SPring-8 large-scale synchrotron radiation facility. The results showed that the accuracy of the contact point detector was not sufficiently high; however, by excluding false positives using semantic segmentation, the detection accuracy improved. We integrated these detection results into a 3D model and successfully estimated the 3D positions of the contact points through clustering. Additionally, we conducted sample-independent experiments, demonstrating that the proposed method achieved detection accuracy equivalent to that of sample-dependent experiments.

## 2 Materials and methods

### 2.1 CT volume data

This study used CT volumes obtained from 11 C*. seticuspe* Gojo-0 samples. Gojo-0 is a self-compatible pure line of *C. seticuspe* (Nakano et al., 2019). The CT volumes of the capitulum inflorescence buds were captured at beamline BL20B2 of the SPring-8 large-scale synchrotron radiation facility. The imaging principles and system are based on existing methods (Uesugi et al., 2010). The X-ray energy was changed from the original method (Yamauchi et al., 2013), whereas other imaging conditions were kept the same as in the original method.

The inflorescence buds of Gojo-0 were pretreated in 90% (v/v) acetone on ice for 20 minutes, followed by a triple wash in phosphate buffer for 15 minutes. Subsequently, they were fixed overnight at 4°C in 4% (v/v) glutaraldehyde dissolved in 0.05 mol*/*L NaPO_4_ buffer. Next, these buds were washed 4 times in phosphate buffered on ice for 15 minutes, followed by post-fixation overnight at 4°C in 1% (v/v) OsO_4_ dissolved in 0.05 mol*/*L NaPO_4_ buffer. After another set of four 15-minute washes with phosphate buffer on ice, the samples were dehydrated using a graded ethanol series on ice. This was followed by three changes in 100% ethanol for 15 minutes, after which they were replaced with t-butyl alcohol. For X-ray CT scanning, the prepared inflorescence samples were freeze-dried overnight (VFD-21, VACUUM DEVICE, Japan). The volume and pixel size of each bud sample and the X-ray energy at the time of capturing are presented in **Table 1**. **Supplementary Figure S1** shows mid-longitudinal sections of the bud samples. ChrGjS601 and ChrGjS600 are in the early growth stage, whereas the others are in the late growth stage.

**Table 1:** The volume size, the pixel size, and the X-ray energy in each bud when the CT data were captured.

Sample ID	growth stage	Volume size (pixel)	Pixel size (µm)	X-ray energy (keV)
ChrGjL601	later	2048×2048×1689	2.75	15
ChrGjL600	later	1836×1850×1778	2.75	15
ChrGjS601	early	1123×1090×920	2.75	15
ChrGjS600	early	926×944×966	2.75	15
Chrgojo01	later	2048×2048×2047	2.70	20
Chrgojo02	later	2048×2048×2047	2.70	20
Chrgojo03	later	2048×2048×2047	2.70	20
Chrgojo04	later	2048×2048×2047	2.70	20
Chrgojo05	later	2048×2048×2047	2.70	20
Chrogjo06	later	2048×2048×2047	2.70	20
Chrgojo07	later	2048×2048×2047	2.70	20

### 2.2 Slice images from CT volume

**Figure 2:**
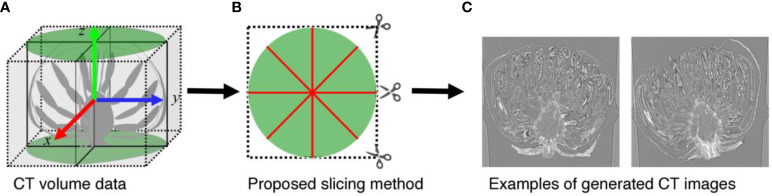
Proposed image slicing method. **(A)** Diagrammatic representation of the volume data. **(B)** shows how **(A)** is sliced when viewed directly above. The green area indicates the area in which the sample is located, and the red line indicates the line to be sliced. **(C)** Examples of slice images.

When analyzing CT volumes, the volumes are sliced into images. As shown in **Figure 1A**, the *C. seticuspe* receptacle has a corn-like rotationally symmetric shape, and the entire bud is also approximately rotationally symmetric about the axis of rotational symmetry of the receptacle, which is shown as the blue dotted line in **Figure 1A**. Therefore, if we slice the CT volume perpendicular to the axis of rotation, the appearance of florets and receptacles in the sliced images will differ significantly depending on the slice position. In machine learning-based image detection methods, the consistency of the object’s appearance greatly influences the task’s difficulty and the detection accuracy. Objects with more consistent appearances are generally easier to detect and yield higher accuracy. Conversely, detection accuracy decreases when the same object has different appearances in an image. Therefore, if images generated by slicing the volume perpendicular to the rotation axis were used, the accuracy of contact point detection would decrease. In this study, we slice the volume along the rotation axis of the bud, as shown in **Figure 2B**. In this method, as shown in **Figure 2C**, even if the slicing position is different, the appearance of the organs on the image remains constant. Furthermore, the proposed slicing method facilitates the detection of contact points because the receptacles and florets are clearly separated on the sliced images.

To generate slice images, two-dimensional (2D) stacked images were transformed from the 3D volume using IMOD software (Kremer et al., 1996). The length, width, and height of the volume are equal to the width and height of the stacked images and the number of stacked images. Slice images were generated in the plane containing the rotational symmetry axis from the 2D stacked images.

In the volume data, the buds were placed in the center of the volume, and the receptacles were oriented upward except for sample Chrgojo02. Thus, we considered the rotation axis of the buds as the z-axis of the volume data, as shown in **Figure 2A**, and generated slice images. We generated 3,600 slice images for each sample by rotating the mid-longitudinal section through the center of the volume data by 0.05° using nearest neighbor interpolation. The parietal of the receptacle of Chrgojo02 was significantly tilted from the center of the volume data. We visualized the volume data using IMOD software, and the rotation axis of the buds was roughly determined from the volume data by visual inspection. The volume data were rotated so that the rotation axis of the bud was set to the z-axis of the volume data, as shown in **Figure 2A**, using IMOD software. Then 3,600 slice images were generated as for the other samples. When we generated slice images from the rotated volume data, some generated images contained no-pixel value areas. These areas were filled with the average of the voxel values of the original volume data to maintain the statistical characteristics of the pixel values in the images. The vertical resolution of the generated images was equal to the height resolution of the volume data, whereas the horizontal resolution was equal to the smaller of the horizontal and vertical volume data resolutions.

In addition to the 3,600 images for each sample, we generated images with the ground truth for training and evaluation. 24 images per sample were generated by rotating the mid-longitudinal sections at intervals of 7.5° from bud samples ChrGjL601, ChrGjL600, ChrGjS601, and ChrGjS600. The ground truth of the contact points between the receptacles and florets and the pixel-wise receptacle regions were manually assigned. For Chrgojo01, Chrgojo02, Chrgojo03, Chrgojo04, Chrgojo05, Chrgojo06, and Chrgojo07, 4 slice images were generated by rotating the mid-longitudinal sections at intervals of 45°. These slice images were also used to determine the ground truths of the contact points and pixel-wise receptacle regions. The CT images are summarized in **Table 2**.

**Table 2:** CT image summary.

Samples	Slice interval (degree)	Images for each sample	Annotation
all	0.05	3600	No
ChrGjL601 ChrGjL600 ChrGjS601 ChrGjS600	7.5	24	Receptacle regions Contact points
Chrgojo01 Chrgojo02 Chrgojo03 Chrgojo04 Chrgojo05 Chrgojo06 Chrgojo07	45	4	Receptacle regions Contact points

### 2.3 Detection of contact points on images

We detected the contact points between the floret and receptacle using machine learning-based object detection. However, the detection results are not perfect. Therefore, we removed detected contact points which are unnaturally far from the receptacle as wrong detection results (i.e., false positives). In the remaining of this section, we present the contact point detection method, the receptacle identification method, and the method used to remove false positives.

#### 2.3.1 Detection of contact points

We used YOLOv5l (You Only Look Once, version 5, large) (Jocher et al., 2022), a representative deep learning-based object detection method, to detect the contact points. The pre-trained YOLOv5l model, which was trained on the Microsoft Common Objects in Context (MS COCO) dataset (Lin et al., 2014), is capable of detecting 80 object categories. To adapt the model for detecting contact points between receptacles and florets, a new dataset was created through manual annotation of bounding boxes around the contact points on sliced images. Subsequently, the pre-trained model was fine-tuned on this dataset, enabling it to detect and localize the contact points.

#### 2.3.2 Semantic segmentation of receptacles

Receptacles are identified using semantic segmentation, which is a computer vision task that aims to categorize each pixel in an image into a specific class or object. In this study, U-Net (Ronneberger et al., 2016), a widely used semantic segmentation model, is employed to detect receptacles. To train the U-Net model, pixel-wise ground truth of receptacles is manually annotated on some sliced images, as described in Section 2.2. The trained model can then be applied to automatically identify receptacles.

#### 2.3.3 Removal of false positives from detector output

Upon analyzing the incorrectly detected contact points, it is observed that they are predominantly located within the floret regions. To address this issue, a simple method is employed to eliminate these false positive detections. The method involves checking whether the bounding boxes of the detected contact points overlap with the receptacle area. If the bounding box of a contact point does not overlap with the receptacle area, the corresponding contact point is considered a false positive and is subsequently removed from the set of detected points. By applying this procedure, the accuracy of contact point detection is improved, as the majority of false positive detections within the floret regions are effectively filtered out. This refinement ensures that the remaining contact points are more likely to represent true contact points between the receptacles and florets.

### 2.4 Identification of 3D position of contact points

To estimate the contact points in 3D space, we integrate the detection results of the contact points on the images. Because the slice images were generated using the method described in Section 2.2, the positions of the generated images in the 3D volume data are known. The contact points in 3D space can be integrated by placing the slice images in 3D space and displaying the detected contact points. It is important to emphasize that the sizes of the contact points are not relevant to the current study. Our primary interest lies in analyzing the spatial distribution and positions of the contact points.

**Figure 3:**
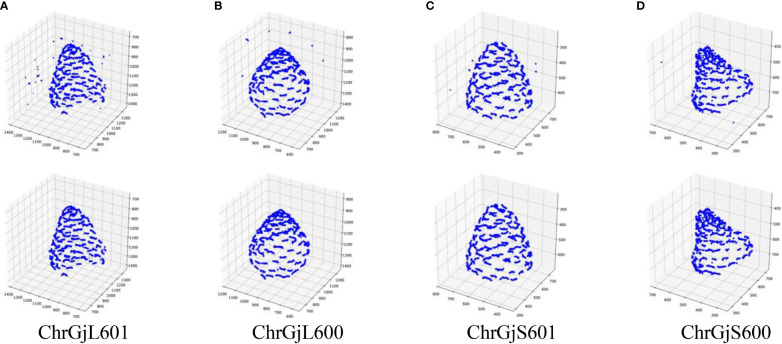
3D integrated contact point detection results for each bud sample: **(A)** ChrGjL601, **(B)** ChrGjL600, **(C)** ChrGjS601, and **(D)** ChrGjS600. The top and bottom rows show the results before and after removing false positives.

Because a contact point has a specific size, it appears on multiple slice images. Therefore, a contact point can be detected on multiple images. Furthermore, the detected positions of the contact points contain errors. Thus, the detected contact points of a contact point are scattered, as shown in **Figure 3**. We need to estimate the position of the contact points using the detection results.

In this study, we employ a clustering algorithm to estimate the positions of contact points from scattered detection results. Clustering is a technique used to divide a set of samples into groups, known as clusters, based on the spatial proximity between the samples. We apply the clustering algorithm to the detection results in 3D space, using the Euclidean distance between the samples as the similarity measure. The clustering algorithm used in this study is the group average method, which is a hierarchical clustering approach. Hierarchical clustering is a bottom-up clustering technique where each sample initially represents a separate cluster. The method then iteratively calculates the similarity between clusters and merges similar clusters into a single cluster. Unlike top-down clustering methods, which divide the sample set into a fixed number of clusters, hierarchical clustering does not require a predetermined number of clusters. This property makes hierarchical clustering suitable for our study, as the number of contact points is unknown. The output of the clustering algorithm is a set of clusters, where each cluster represents a group of detection results corresponding to a single contact point. To estimate the position of each contact point, we calculate the average of the detection results within each cluster. The number of clusters obtained from the clustering algorithm is equal to the number of contact points in the biological structure. By applying the group average hierarchical clustering algorithm to the scattered detection results, we can effectively estimate the positions of the contact points without prior knowledge of their number. This approach allows us to analyze the spatial distribution of the contact points in the biological structure, providing valuable insights into the underlying mechanisms and functions.

The group average method calculates the similarity between two clusters as the average of all pairwise distances between the samples in the two clusters. Let *A* and *B* be two clusters, and let | · | denote the number of samples in a cluster. Let x and y be the coordinates of the detected contact points which separately belong to clusters *A* and *B* in 3D space. Let d(x,y) be the distance between two samples, specifically the Euclidean distance between two detected contact points. The group average can then be described by the following Equation 1:


(1) $$\frac{1}{\left|A\right|\left|B\right|}\sum_{\text{x}\in A}\;\sum_{\text{y}\in B}d\left(\text{x},\text{y}\right).$$

To obtain appropriate clustering results, it is necessary to set termination conditions for the hierarchical clustering process. In this study, the termination condition is defined such that the clustering process stops when the smallest value of the group average between any two clusters exceeds a predetermined threshold.

## 3 Experiments

In the experiments, we first evaluated the contact point detection method. Next, we evaluated the estimation of the 3D position of the contact points. In these evaluations, we performed a sample-dependent evaluation: the training and evaluation data were derived from the same sample, but the training and test data were not identical. Furthermore, we evaluated the estimation of the 3D position of the contact points in a practical scenario; the evaluation data were derived from other samples different from those that generated training data for the contact point detector and receptacle segmentation. In the following section, we describe the experimental setup and evaluation metrics and show the results of each evaluation.

### 3.1 Evaluation of the contact point detection method

We calculated the accuracy of the contact point detection method. As mentioned in Section 2.3, the contact point detection method comprises three elementary methods: contact point detector and receptacle segmentation, and false positive removal of the detector. To evaluate each of the elementary methods, we used method-specific metrics.

#### 3.1.1 Contact point detector

To train and evaluate the contact point detector, we used four CT volumes: ChrGjL601, ChrGjL600, ChrGjS601, and ChrGjS600. Twenty-four slice images were generated from each volume, and the ground truths were manually assigned in each CT volume, as described in Section 2.2. Because the size of the contact point region is smaller than the size of the slice images, the contact point may not be detected. Therefore, we cropped the slice images so that they included the entire receptacle. Because the position and size of the receptacles are different for each CT volume, we changed the cropping region according to the CT volumes. **Supplementary Table S1** shows the coordinates of the upper left and lower right corners of the crop regions on the slice images.

The evaluation was performed in a sample-dependent scenario. We trained the model on each bud sample and evaluated it using the same bud sample for training. We evaluated the detection accuracy using leave-one-out (LOO) cross-validation on the cropped images. In LOO cross-validation, a datum is selected from a dataset, and the model is trained using the remaining data in the dataset. The trained model is evaluated using the selected datum. This process is repeated until all data in the dataset have been selected for evaluation. The average accuracy of all evaluations is calculated as the model accuracy.

The specific uses of the training and evaluation data are as follows. For each bud sample, 1 of the 24 images was selected for accuracy evaluation, and the remaining 23 images were used for training. Because the 23 images were too small to train the model, we augmented the images to 2,300 and used them for training. The trained model was evaluated on the selected image. We repeated the process 24 times and calculated the average accuracy as the performance of the contact point detector.

The details of how we augmented 23 images to 2,300 are as follows. 23 images were flipped from left to right to generate 23 images. We applied geometric transformations, including translation, rotation, and scaling, to the original cropped and flipped images, bringing the total number of images to 2,300. The specifications of each geometric transformation are as follows: Translation moved the image vertically or horizontally by a distance of 10% or less of its height or width. Rotation was a clockwise or semiclockwise rotation around the image center at a random angle between 0°and 20°. Scaling was performed by enlarging the image at a random scale between 0.95 and 1.05. The missing areas caused by these augmentation processes were filled with the mean pixel values of the original cropped image so that the size of the augmentation image was the same as that of the original crop image.

For model training, we used Adam as the optimization algorithm for network training. The batch size was set to 16, and 25 epochs were trained (the loss curve is not provided). The initial training rate was set to 10^−3^, and after 25 epochs, it was linearly reduced using the scheduler to 10^−4^. During training and verification, the input images were resized to 640 × 640 pixels while keeping the aspect ratio. The longer side of the image was set to 640 pixels. The blank space resulting from resizing was filled with the average pixel value of ImageNet (Deng et al., 2009).

We used average precision (AP) as an evaluation metric. AP is a model prediction metric that represents the accuracy of the model outputs. Let p(⋅) and *r* be the precision and recall of the model, respectively, and AP is calculated using the following Equation 2:


(2) $$\text{AP}=\int_{0}^{1}p\left(r\right)dr.$$

Because AP is calculated on the basis of the accuracy of the model output, criteria are required to determine whether the model output is correct when evaluating object detection methods, such as those used for contact point detection. Intersection over union (IoU) is generally used as an object detection criterion. IoU is a criterion that indicates the extent to which the object region predicted using the model and the ground truth overlap. Let *P* and *G* be a set of image pixels in the predicted and ground truth object regions, respectively. The IoU between the prediction and ground truth is defined by the following Equation 3:


(3) $$\text{IoU}\left(P,G\right)=\frac{\left|P\cap G\right|}{\left|P\cup G\right|}.$$

If IoU is greater than the threshold value, the object is correctly detected.

In the experiment, we calculated three types of AP using the same method as that in the MS COCO dataset (Lin et al., 2014): AP50, AP75, and AP(0.5:0.95). AP50 and AP75 are the AP values when the threshold of IoU is 0.5 and 0.75, respectively. AP(0.5:0.95) is the AP value when the IoU threshold changes from 0.5 to 0.95 with an interval of 0.05.

The implementation of the AP calculation was performed as follows:

1. The detection results were sorted by the confidence value output by the detector.
2. The IoU for each detection result was calculated. If the IoU was greater than the threshold, the detection results were considered correct. Cumulative precision and recall were calculated from the thresholding results.
3. The AP value was calculated using interpolation and approximation, as in the MS COCO dataset.

#### 3.1.2 Receptacle segmentation

Four bud samples, ChrGjL601, ChrGjL600, ChrGjS601, and ChrGjS600, were used to evaluate receptacle segmentation, as in the case of contact point detection. The slice images used to evaluate receptacle segmentation were also the same images used to evaluate contact point detection. The receptacle regions were manually assigned to the slice images, and the receptacle region was used for model training and evaluation. No cropping was performed because the receptacle regions to be detected were sufficiently large for the slice images.

We evaluated the segmentation accuracy using LOO cross-validation, as described in Section 3.1.1. The Dice coefficient, which is commonly used in segmentation evaluation, was used as the evaluation metric. The Dice coefficient DCS(*P,G*) of the predicted and ground truth object regions *P* and *G* is given by the following Equation 4:


(4) $$\text{DSC}\left(P,G\right)=\frac{2\left|P\cap G\right|}{\left|P\right|+\left|G\right|}.$$

The Dice coefficient becomes large when the overlap between the two regions is large.

The data augmentation applied was the same as that in Section 3.1.1; 24 slice images were generated for each bud sample. 23 slice images were augmented to 2,300 by left and right inversion and data augmentation procedures. The remaining image was used for evaluation.

We used U-Net as the segmentation model. We replaced the encoder section of U-Net with ImageNet pretrained VGG16 (Simonyan and Zisserman, 2015) and used it in the experiment. We used Adam as the optimization algorithm. The training rate, batch size, and number of epochs were set to 10^−4^, 32, and 25, respectively. The size of the images used for the experiments was normalized to 512 × 512 pixels.

#### 3.1.3 Removal of contact point false positives

We evaluated the false positive removal method in a bud sample-dependent scenario. Contact point detection and receptacle segmentation were performed on 3,600 slice images of each bud sample. The detector and segmentation model were trained on 2,400 images from each bud sample. The 2,400 images were augmented from the 24 images using the ground truths described in Section 2.2. All other experimental settings were the same as those described in Sections 3.1.1 and 3.1.2.

#### 3.1.4 Estimation of the 3D position of contact points

After removing false positives, we integrated the detection results and estimated the 3D position of the contact points using hierarchical clustering. We used the images and detection results obtained in Section 3.1.3 in the experiment.

When the group average between clusters was greater than or equal to threshold *d*, cluster merging was terminated. We changed *d* to 1, 10, 20, 30, 40, and 50 and investigated the number of clusters and the clustering results in 3D integrated data.

#### 3.1.5 Accuracy evaluation of the proposed method

To evaluate the accuracy of the integration of the detected contact points, we conducted further experiments. In these experiments, we evaluated three aspects related to 3D position estimation: outlier removal, image slicing method, and accuracy of the distance between the contact points. In these experiments, we evaluated the 3D coordinate of the contact points estimated by the proposed method using the sample ChrGjL600 with the clustering threshold of 50 as the ground truth. This is because it is difficult to obtain the ground truth of the contact points for the bud samples.

First, we evaluated the effectiveness of outlier removal. We integrated the 2D contact point detection without false positive removal explained in Section 2.2 for ChrGjL600. Then, we calculated the error between the estimated detected points and the ground truth. We considered the correspondence point of an estimated contact point as the nearest contact point on the ground truth and calculated the error.

Next, we used a simulation to evaluate the influence of the image slicing method and the accuracy of the distance between the contact points. Since slice images are generated as shown in **Figure 2B**, the interval between images changes depending on the distance from the axis. Therefore, we conduct simulation experiments to evaluate whether this difference in image interval affects the accuracy of contact point estimation. We generated slice images from the estimation data; the coordinate of the contact points on the slice images was known. We considered the generated slice images as the slice images after applying contact point detection and outlier removal, and then performed 3D integration and clustering with the threshold of 50 on the generated slice images. We evaluated the estimation accuracy by the error between the estimated contact points and the ground truth. In the proposed image slicing method, the sampling rate depends on the distance from the rotation axis of the buds, and this difference in sampling rate may affect the 3D position estimation results. To evaluate the effect of the proposed image slicing method, we plotted the errors with the horizontal axis as the distance from the rotation axis of the buds and the vertical axis as the error.

We also evaluated the distance between the contact points, which we plan to use in future analyses mentioned in Section 1. We calculated the distance between each contact point and its closest contact point, and calculated the error between them and the distance calculated using the ground truth.

### 3.2 3D contact point estimation in a practical scenario

Previous experiments were conducted using sample-dependent scenarios. However, considering the practical use of this method, a sample-independent scenario is preferable because it reduces the time and effort required for annotation. We then evaluated the proposed method in the sample-independent scenario.

We used ChrGjL601, ChrGjL600, ChrGjS601, and ChrGjS600 for training and Chrgojo01, Chrgojo2, and Chrgojo03 for validation. Chrgojo04, Chrgojo05, Chrgojo06, and Chrgojo07 were used for testing. We used the labeled slice images from previous experiments as training data, i.e., 24 images for each bud sample, totaling 96 images. The regions shown in **Supplementary Table S1** were cropped when the images were used for contact point detection.

96 labeled slice images were augmented to 9,600 images for training. We used appropriate augmentation methods for the contact point detector and receptacle segmentation. To train the contact point detector, we used RandAugment, one of the augmentation methods proposed by Cubuk et al (Cubuk et al., 2020). The parameters *n* and *m* of the RandAugment, which represent the number of augmentation transformations to apply sequentially, and the magnitude for all the transformations, were set to 4 and 25, respectively. For receptacle segmentation, the augmentation procedure was the same as that described in Section 3.1. Other training configurations, such as the training algorithm, batch size, and learning rate, were the same as those described in Section 3.1. We choose a model that outputs the highest accuracy on the validation dataset among all training epochs for evaluation. Using the outputs of the selected models, we performed false positive removal and 3D point estimation.

## 4 Results

**Figure 4:**
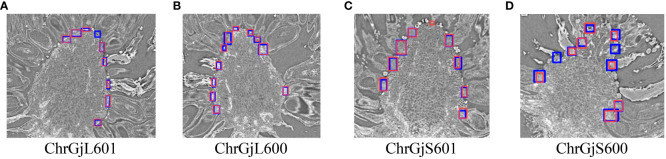
Example of the detection results for the contact point between florets and receptacle for each bud sample: **(A)** ChrGjL601, **(B)** ChrGjL600, **(C)** ChrGjS601, and **(D)** ChrGjS600. The blue rectangles indicate the ground truth of the contact points. The red bounding boxes indicate the detection results. The independent blue rectangle in **(A, D)** and independent red rectangle in **(C)** indicate failure in detection (false negative) and wrong detection (false positive), respectively.

We show the evaluation results of contact point detection. **Table 3** shows the AP50, AP75, and AP(0.5:0.95) values for each bud using LOO cross-validation. **Figure 4** shows the example detection results and ground truth for each bud sample.

**Table 3:** Contact point detection results.

Sample ID	AP(0.5:0.95)	AP50	AP75
ChrGjL601	0.4218	0.8898	0.3341
ChrGjL600	0.5167	0.9382	0.4908
ChrGjS601	0.5552	0.9510	0.5872
ChrGjS600	0.3441	0.7791	0.2283

**Figure 5:**
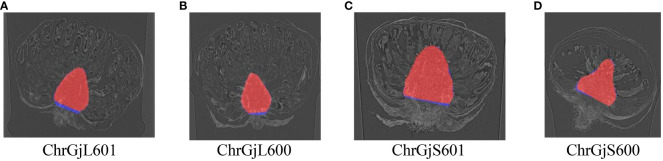
Examples of receptacle segmentation results overlaying the ground truth for each bud sample: **(A)** ChrGjL601, **(B)** ChrGjL600, **(C)** ChrGjS601, and **(D)** ChrGjS600. The red regions indicate the segmentation results, and the blue regions indicate the ground truth that does not overlap.

Then, receptacle segmentation results were presented. The Dice coefficients of receptacle segmentation for each bud sample are shown in **Table 4**. Examples of the segmentation results are shown in **Figure 5**.

**Table 4:** Dice coefficients of receptacle segmentation for each bud sample.

Sample ID	Dice coefficient
ChrGjL601	0.9761
ChrGjL600	0.9810
ChrGjS601	0.9745
ChrGjS600	0.9640

The false positives of the contact points were removed based on the receptacle segmentation results. The 3D integrated detection results before and after removing false positives were plotted on 3D coordinates, as shown in **Figure 3**.

**Figure 6:**
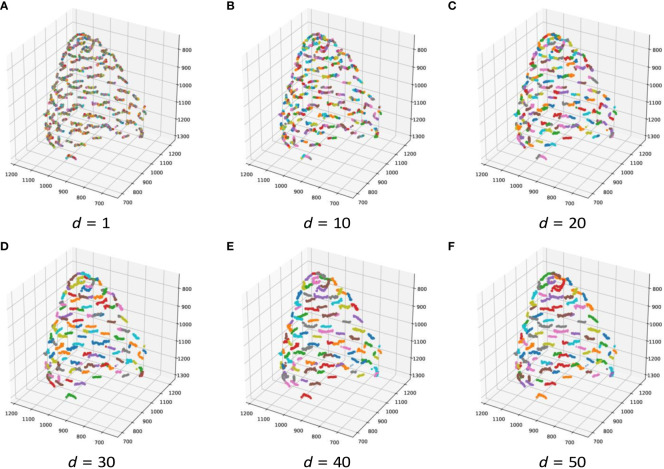
Contact point clustering results when *d* changes in 3D for ChrGjL601. Because of the large number of clusters, it was difficult to color all clusters with different colors. Therefore, 10 different colors were used to paint the clusters so that neighboring clusters would not have the same color.

**Figure 7:**
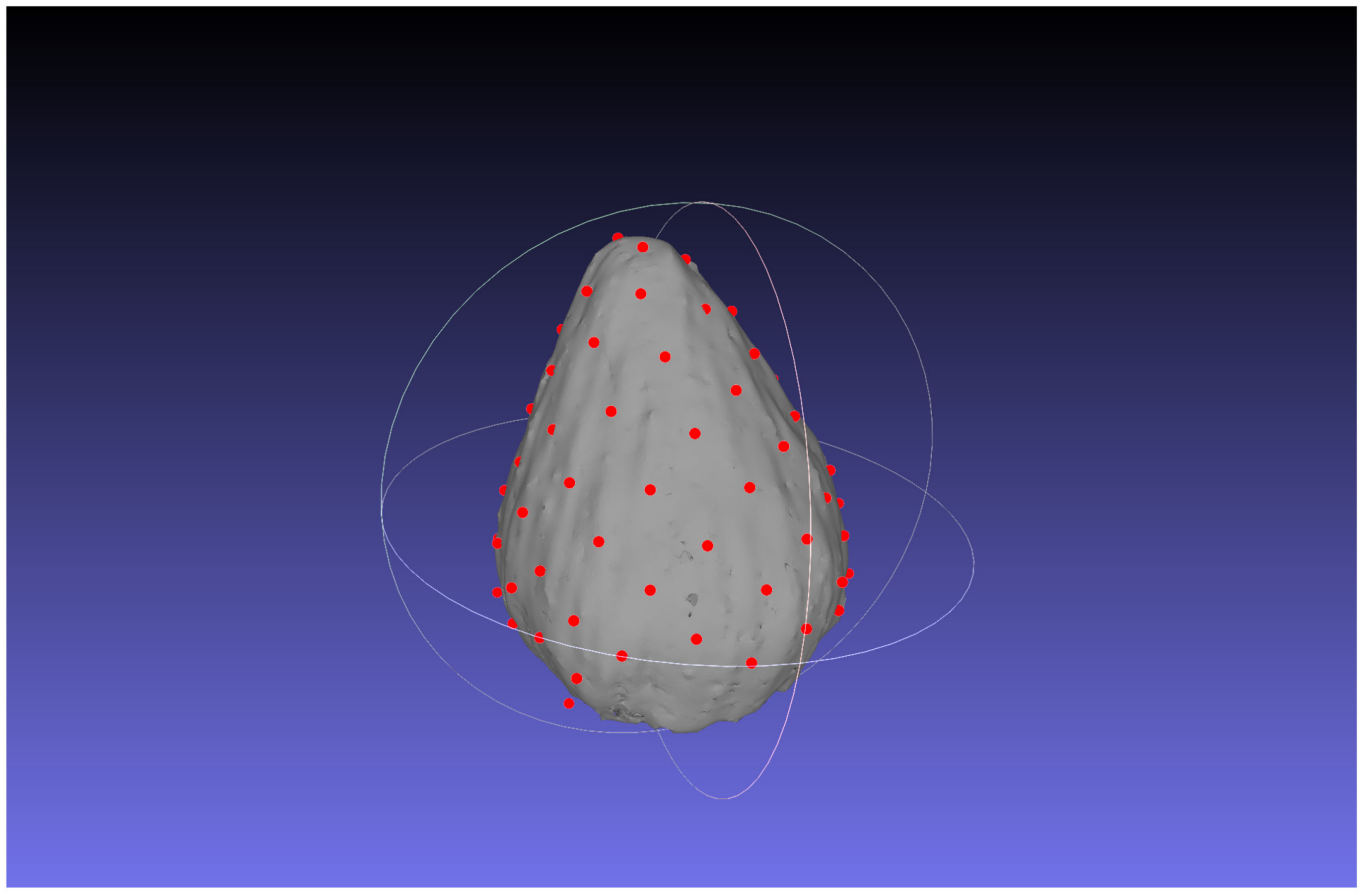
Contact point estimation results for ChrGjL600 at *d* = 50 with the receptacle. The red dots represent the estimated contact points, and the white object represents the reconstructed receptacle from the segmentation result.

The estimation results of the 3D position of the contact points are as follows. The number of clusters when *d* changed in each bud sample is shown in **Table 5**. The clustering results in 3D data when *d* was changed are shown in **Figure 6**; **Supplementary Figures S2–S4**. The dots show the detected contact points, and each cluster is colored differently. We also plotted the estimated contact points, which are the means of the clusters, with the receptacle at *d* = 50 for ChrGjL600, as shown in **Figure 7**. To better illustrate the 3D data, see a video of this rotated plot in **Supplementary Material**. The same video has been uploaded to YouTube (https://youtu.be/w9TriAqQan4). The receptacle was drawn by integrating the contours of the segmentation results of the slice images in 3D and reconstructing the surface using the ball-pivoting algorithm (Bernardini et al., 1999). Laplacian smoothing was then applied to the reconstructed surface. OpenCV (https://opencv.org), an image processing library available from Python, was used for contour detection. MeshLab (Cignoni et al., 2008) was used for the ball-pivoting algorithm, Laplacian smoothing, and rendering.

**Table 5:** Contact point estimation results.

Sample ID	Threshold *d*						Ground truths
	1	10	20	30	40	50	
ChrGjL601	6606	428	205	132	103	101	108
ChrGjL600	6589	444	208	126	97	96	95
ChrGjS601	4955	329	145	99	95	91	93
ChrGjS600	4098	307	134	104	95	64	100

**Figure 8:**
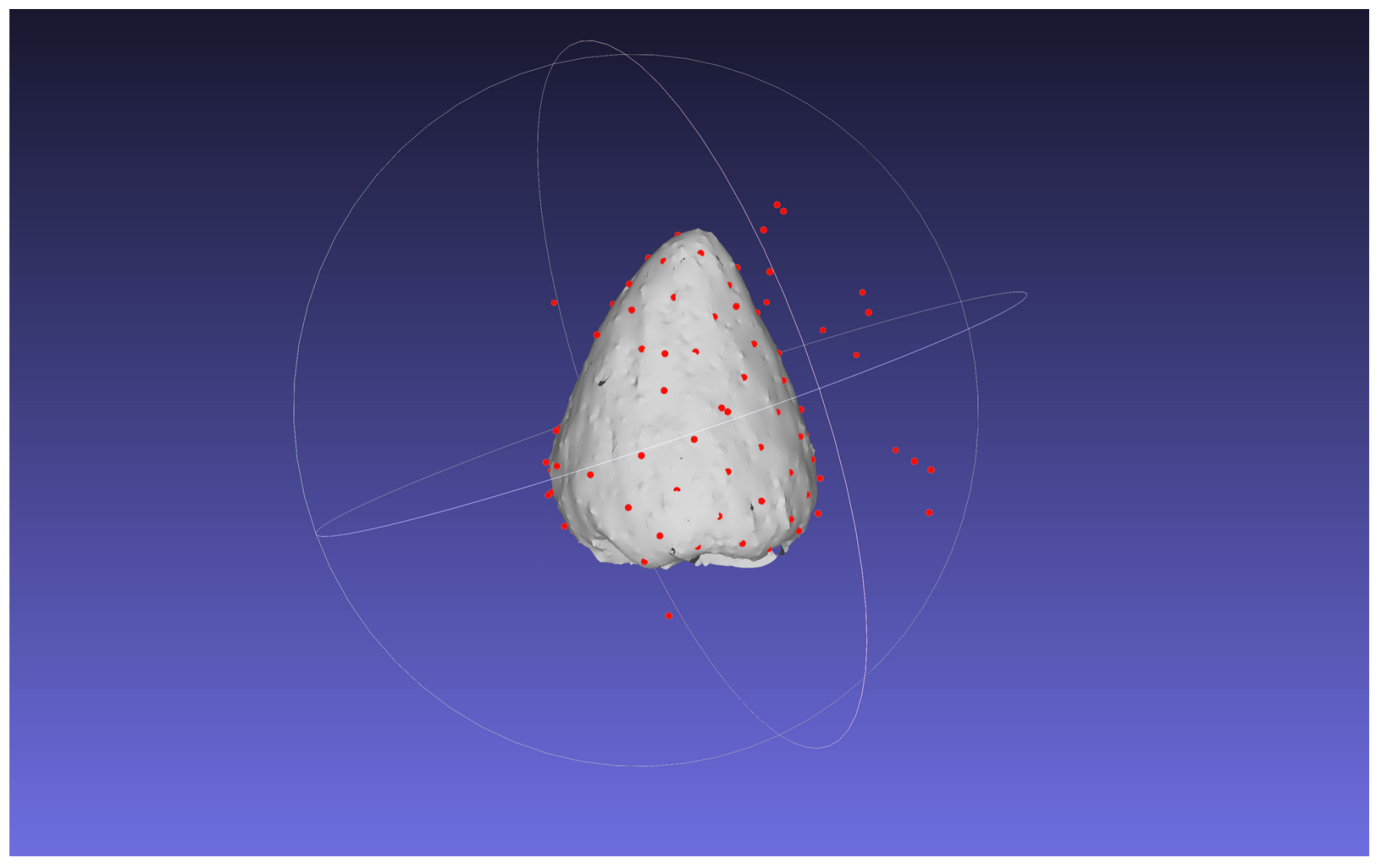
Contact point estimation results for ChrGjL600 at *d* = 50 without false positive removal.

**Figure 9:**
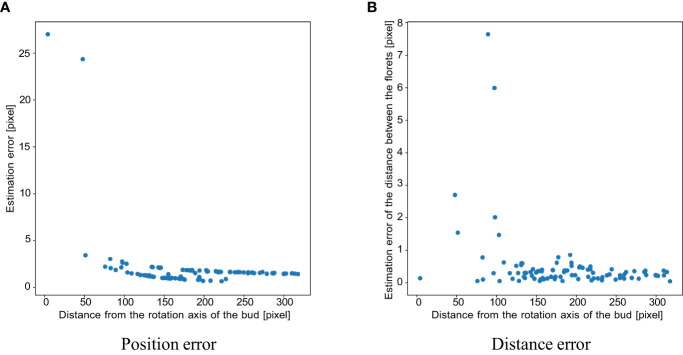
Scatter plots of **(A)** position and **(B)** distance estimation error for each contact point in the simulation experiment, where the horizontal axis is the distance of the contact point from the axis of rotation of the bud.

The results of the verification experiment on the contact detection accuracy in Section 3.1.5 are as follows. The mean and standard deviation of the contact point estimation error without outlier removal were 14.843 and 2690.05, respectively. **Figure 8** shows the estimated position of the contact points in 3D without outlier removal. To evaluate the influence of the image slicing method, **Figure 9A** shows the plot of the error with the horizontal axis as the distance from the rotation axis. The mean and standard deviation of the errors are 1.881 and 12.588, respectively. The mean and standard deviation of the errors for the distance between the florets are 0.454 and 1.040, respectively. **Figure 9B** shows the scatterplot where the horizontal axis is the distance from the rotation axis of the bud and the vertical axis is the absolute distance error. From the experimental results, false positives removal has a significant effect on improving the accuracy of estimating the 3D position of the contact points. Also, the closer to the axis, the worse the accuracy of estimating the 3D position of the contact points.

Finally, we show the result of 3D contact point estimation in a practical scenario. **Supplementary Tables S2**, **S3** show the APs of contact point detection and the Dice coefficients of receptacle segmentation. **Supplementary Figures S5–S11** show examples of contact point detection results, receptacle segmentation results, detection results after the removal of false positives, and clustering results of 3D contact points for Chrgojo04, Chrgojo05, Chrgojo06, and Chrgojo07, respectively.

## 5 Discussion

The proposed method could estimate the contact points between florets and receptacles as shown in **Figure 7**. The 3D plot allows us to analyze the inflorescence in 3D, which could not be done using only 2D images. In the following section, we discussed the error of the estimated contact points by the proposed method, and the experimental results.

### 5.1 Contact point estimation error

We analyze the potential error in estimating the contact point position as a cluster center in two aspects, as shown in **Supplementary Figure S12A**: radial and tangent directions.

#### 5.1.1 Error in the radial direction

The error in the radial direction corresponds to that within a sliced image. **Table 3** shows that the AP50 score for the detection of contact points is high, while the AP75 and AP(0.5:0.95) scores are low. This result indicates that many contact points are detected with IoUs between 0.5 and 0.75. Since an IoU of 0.5 corresponds to the situation in which the ground truth area and the detected area overlap by 2*/*3, a slight shift in the detection position of the contact point causes a significant decrease in IoU, as shown in **Figure 4**. The primary cause of this phenomenon is that the detection targets are small and lack sufficient detailed appearance information to distinguish them from the background and similar objects [e.g., Bai et al. (2018)]. However, in this experiment, the contact point can still be detected even if the IoU value for contact position detection is low, as long as it falls within the detection area.

Furthermore, any detection errors can be corrected during the 3D integration process. This phenomenon is explained as follows by the law of large numbers. Let *N* be the number of 2D images that contain a contact point; imagine that a cluster consists of approximately *N* points in **Figure 6**. For simplicity, let us assume a one-dimensional case where errors in the detected positions of the contact points appear only horizontally, while the same argument can be applied to a general two-dimensional case. Suppose that the amount of displacement, say *x*, is a random variable, and $x_{1},x_{2},\ldots ,x_{N}$ denote concrete values drawn from the distribution. Then, regardless of its distribution, the average displacement, $\frac{1}{N}\sum_{i=1}^{N}x_{i}$ , is expected to become close to zero with a large *N*. In the extreme case, it is expressed as in Equation 5:


(5) $$\underset{n\to \infty }{\lim}\frac{1}{N}\sum_{i=1}^{N}x_{i}\to 0.$$

However, even with a relatively large *N* such as in our experiment, we can expect that the average displacement is close to zero regardless of the amount of displacement. In a similar argument, we can also expect that the effect of failure in detecting contact points is minimized.

The 3D position of the contact point is estimated by taking the average of the detected positions in each cluster. Therefore, even if there is an error in the detection of a contact point in the 2D image, the above discussion suggests that the error does not significantly affect the accuracy of the 3D position estimation.

#### 5.1.2 Error in the tangent direction

The error in the tangent direction corresponds to that across the sliced images, which also includes the arbitrariness of the image slicing selection. **Supplementary Figure S12B** illustrates a case where the estimated cluster center does not move regardless of the slicing positions of the images. In contrast, **Supplementary Figure S12C** illustrates a case where the estimated cluster center shifts with slight changes in the slicing positions. In the latter scenario, the maximum value of the tangential error will be the distance between two adjacent images, whose distance grows proportionally to the distance from the rotation center of the CT volume. So far, we have assumed that no contact point detection errors occur. If detection errors do occur, it is obvious that the estimated cluster center will shift. Let us consider the scenario of failing to detect each contact point in each sliced image. The point farthest from the cluster center has the greatest effect on the displacement of the estimated cluster center, which the detection errors of other points have a smaller impact. However, given the high detection accuracy shown in **Table 3**, such errors are rare. Therefore, the positions of the estimated cluster centers are considered to be reasonably precise.

Taking ChrGjL601 as an example, let us calculate the possible maximum error. As shown in **Table 1**, the width and height of the CT volume are each 2048 pixels. Since each side corresponds to 90 degrees and the sliced images exist at every 0.05°, each side is divided into 90/0.05 = 1800 sliced images. Therefore, the possible maximum error is given as 2048*/*1800 ∼ 1.14 pixels. Considering that each pixel represents 2.75 µm, this corresponds to a deviation of up to approximately 3.13 µm at the edge of the CT volume. According to **Figure 6**, clustering is most successful when *d* = 40, so a reasonable distance between contact points is about 40 pixels. The contact points exist near the center of the image, so the error is smaller, so we believe that the error caused by the proposed method is within the acceptable range.

### 5.2 Sample difference in contact point detection

**Figure 10:**
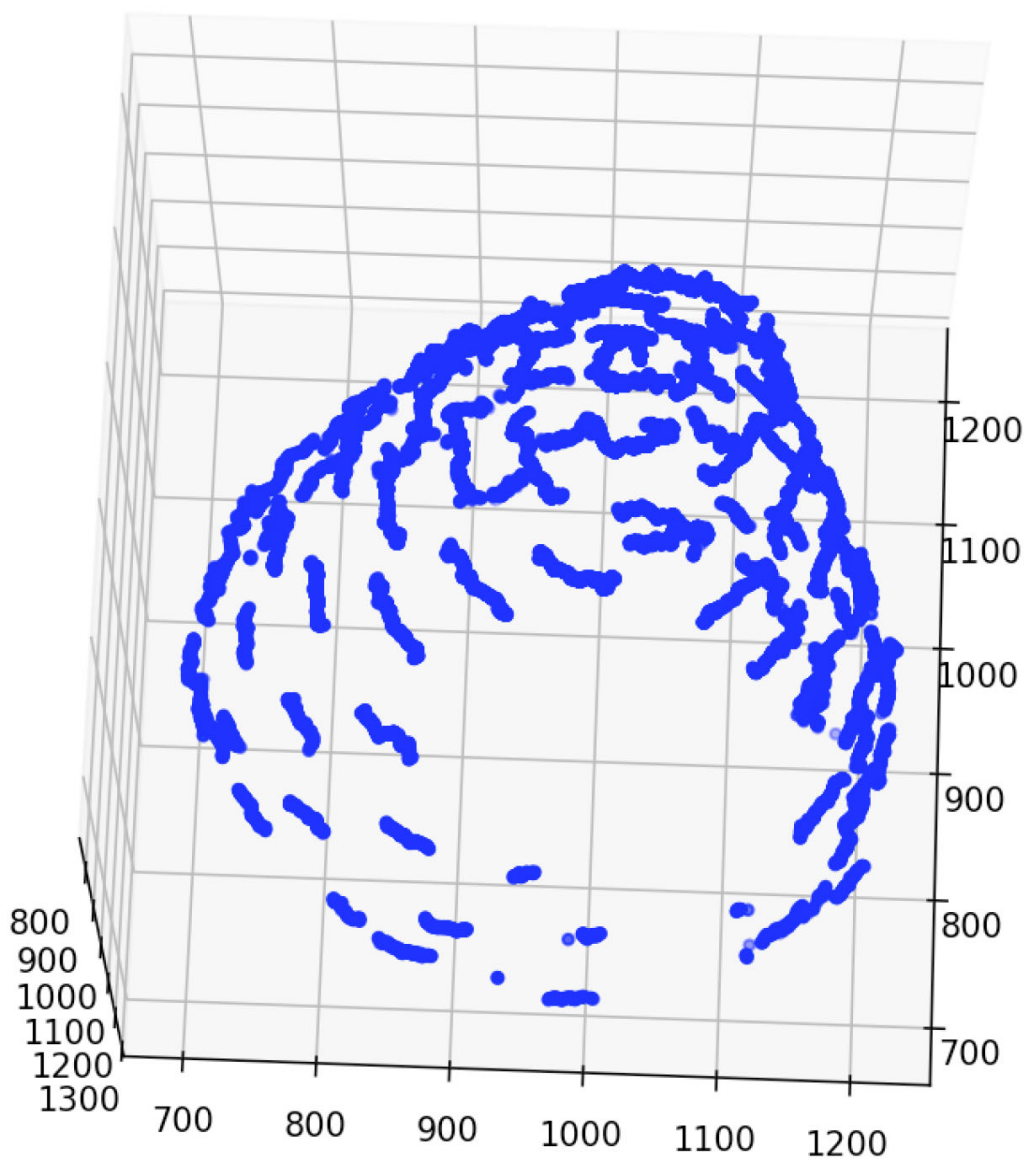
Regions in which contact points were not detected for ChrGjL601.

As presented in **Table 3**, ChrGjS600, which was at an early growth stage, had lower contact detection accuracy than the other bud samples. This is because the receptacle has not yet developed in the early growth stage, and the boundary between the floret and receptacle regions is unclear. As shown in **Figure 10**, the 3D visualization of contact points for ChrGjL601 revealed certain regions where contact points were not detected. This phenomenon did not occur in any other sample. Therefore, this phenomenon is not caused by the sample treatment process, but by some impact applied only to this sample. In contrast, this result shows that the accuracy of the detector is so high that it does not detect a contact point if there is a small gap between the petal and receptacle.

### 5.3 Receptacle segmentation

Regarding receptacle segmentation, the LOO cross-validation accuracy was high, as presented in **Table 4**. This is because of the slicing method proposed in Section 2.2. The receptacles were located in the center, and their shape and texture were consistent in the slice images generated using the proposed method. Therefore, receptacle detection has become a simple task, and we can achieve high accuracy in receptacle segmentation.

### 5.4 False positive removal of contact point detection

In **Figure 3**, false positives from contact point detection are removed from the detection results. Because the appearance of the contact point is similar to that of the floret area, contact point detection incorrectly detects similar receptacle areas. Because object detection detects the target object based on its visual similarity in an image, it is difficult to reduce false positives when the image contains regions that have a similar appearance to the target object. Although contact point detection had false positives, the accuracy of the receptacle segmentation results was high. Then, by combining the results of contact point detection and receptacle segmentation, it was possible to remove false positives and detect contact points with high accuracy. **Figure 8** shows the result of 3D integration of the contact point detection results without false positive removal. Some of the contact points are far from the receptacle, indicating that the estimation results are clearly incorrect. This indicates that false positive removal is indispensable for accurate 3D position estimation.

### 5.5 Clustering for integrating detection results in 3D

**Table 5** demonstrates that, when estimating the 3D position of the contact points, adjusting the threshold *d* (which serves as the termination condition) enabled the acquisition of clusters closely approximating the original contact points. Because hierarchical clustering does not set the termination condition automatically, it is necessary to adjust the termination condition manually. As shown in **Figure 3**, a set of detected contact points from a contact point was scattered, and each set of detected contact points was separated from each other. Therefore, the threshold can be adjusted by making each set of detected contact points a cluster in 3D space. For instance, **Figure 6** illustrates the results of contact point clustering for sample ChrGjL601 using various values of the distance threshold *d*. Ideally, each cluster should be assigned a unique color. However, due to the large number of clusters, we limited the color palette to 10 colors and assigned different colors to neighboring clusters. When a single blob contains multiple colored clusters, it indicates over-segmentation when *d* = 1,10,20,30, as shown in **Figures 6A–D**. In contrast, when multiple blobs are assigned the same color, it suggests over-grouping of clusters, as observed when *d* = 50, as shown in **Figure 6F**. Therefore, the appropriate *d* value was determined to be 40, as shown in **Figure 6E**, because each blob was clustered into a single cluster. Thus, appropriate clustering parameters can be determined by plotting the clustering results in 3D, as shown in **Figure 6**, and verifying that each blob is classified into a single cluster by visual inspection. As shown in **Supplementary Figures S2–S4**, the other bud samples, along with ChrGjL601, can determine the appropriate values for the parameter *d*.

### 5.6 Simulation experiment in 3D contact point estimation

From **Figure 9A**, the contact points that were close to the rotation axis have larger errors than the contact points that were far from the axis. This is due to the clustering error, not the sampling density. Because the shape of the receptacle was conical, the contact points that were close to the rotation axis were placed at the top of the receptacle. The contact points were densely placed on the top of the receptacle. Therefore, the clustering fails, and the error between the ground truth and the estimation results becomes larger. The distance between the contact points also has the same tendency as the estimation error. From **Figure 9B**, distance errors also tend to be larger for contact points closer to the axis of bud rotation, i.e. closer to the apex of the receptacle. The clustering results depend on the value of the parameter *d*: if *d* is small, the number of clusters will increase. In the experiment, when we applied the clustering method with the same *d* for all contact points, the clustering method would fail on the top of the receptacle where the contact points were dense. One of the solutions to improve the clustering result is to apply the clustering method while changing the parameter *d* adaptively. If we apply the clustering method with smaller *d* for the top of the receptacle, more accurate clustering results would be obtained. It would lead to more accurate contact point estimation and be able to perform pattern analysis of floret phyllotaxis.

The average distance between contact points is about 50 pixels. As shown in **Figure 9B**, the error in this distance is less than 1 pixel, providing sufficient accuracy for subsequent analyses that rely on these measurements. However, **Figure 9B** also shows that the error in the distance between contact points near the rotation axis, that is the contact points close to the apex, can reach up to nearly 8 pixels. This level of error is significant enough to impact subsequent analyses. Therefore, improving the accuracy of the vertices by adjusting their clustering parameters could lead to more precise future analyses.

### 5.7 Sample-independent scenario

As shown in **Supplementary Tables S2**, **S3**, the detection accuracy of the contact point detection and receptacle segmentation in the sample-independent scenarios (presented in Section 3.2) was comparable to that in the sample-dependent scenarios (presented at the beginning of Section 3). The false positive removal and contact point clustering were also successful, as shown in **Supplementary Figures S7–S11**. Therefore, the proposed method is useful for detecting contact points in real-world scenarios.

In **Supplementary Table S2**, Chrgojo07 shows lower accuracy compared to other samples, likely due to texture-level differences in the contact points’ appearance. Neural networks are sensitive to texture variations, which can influence detection results (Hermann et al., 2020). While Chrgojo07 may seem similar to other samples at first glance, subtle texture differences may have caused detection failures. Increasing the number of training samples could potentially improve its accuracy.

### 5.8 Limitation of the proposed method

A limitation of this study is that we could not experimentally prove the applicability of the proposed method when the variety of the scanned flower changes. In our experiments, we used only CT images of *C. seticuspe*. However, when applying the proposed method to other plants, it may fail due to variations in receptacle shape and floret arrangement.

## 6 Conclusion

In this study, to understand the 3D structure of the *C. seticuspe* bud, we collected the 3D data by CT and detected the contact points between the florets and the receptacle from the slice images using image recognition technology and displayed them in 3D space. To facilitate the contact point detection task, we proposed a new image slicing method for CT volumes such that all slice images have a similar appearance. Furthermore, we segmented the receptacles and removed detected points not on the receptacles as false positives. When the results of contact detection on the slice images were integrated into 3D space, the contact points were not uniquely determined because of the contact point size. We used a clustering method to estimate the position of the contact points. The experimental results showed that the proposed method estimated the contact points when the clustering parameter was set appropriately. The results also indicated that the proposed method could estimate the contact points even when the training and test images were generated from different bud samples.

A future task is to automate the clustering parameters, which are currently determined manually. We also plan to develop a mathematical model of the position of the contact point between the receptacle and florets based on the estimation results.

## Data availability statement

The labeled data for this study can be found in the Figshare https://doi.org/10.6084/m9.figshare.25388434. The raw data supporting the conclusions of this article will be made available by the authors, without undue reservation.

## Author contributions

SM: Formal analysis, Software, Validation, Visualization, Writing – original draft. YU: Conceptualization, Funding acquisition, Methodology, Supervision, Writing – original draft, Writing – review & editing. TK: Conceptualization, Data curation, Supervision, Writing – review & editing. MI: Methodology, Supervision, Writing – review & editing. TN: Data curation, Writing – review & editing. DY: Data curation, Writing – review & editing. IK: Data curation, Writing – review & editing. YM: Data curation, Writing – review & editing. MH: Data curation, Writing – review & editing. KU: Data curation, Writing – review & editing. KK: Resources, Writing – review & editing.
