# Supplementary material for: CT image-based 3D inflorescence estimation of Chrysanthemum seticuspe
Source: Front Plant Sci. 2024 Jul 29;15:1374937. doi: 10.3389/fpls.2024.1374937 (PMC11317900; doi:10.3389/fpls.2024.1374937)
Supplement: Supplementary file 1 [file DataSheet_1.pdf]

Supplementary Material

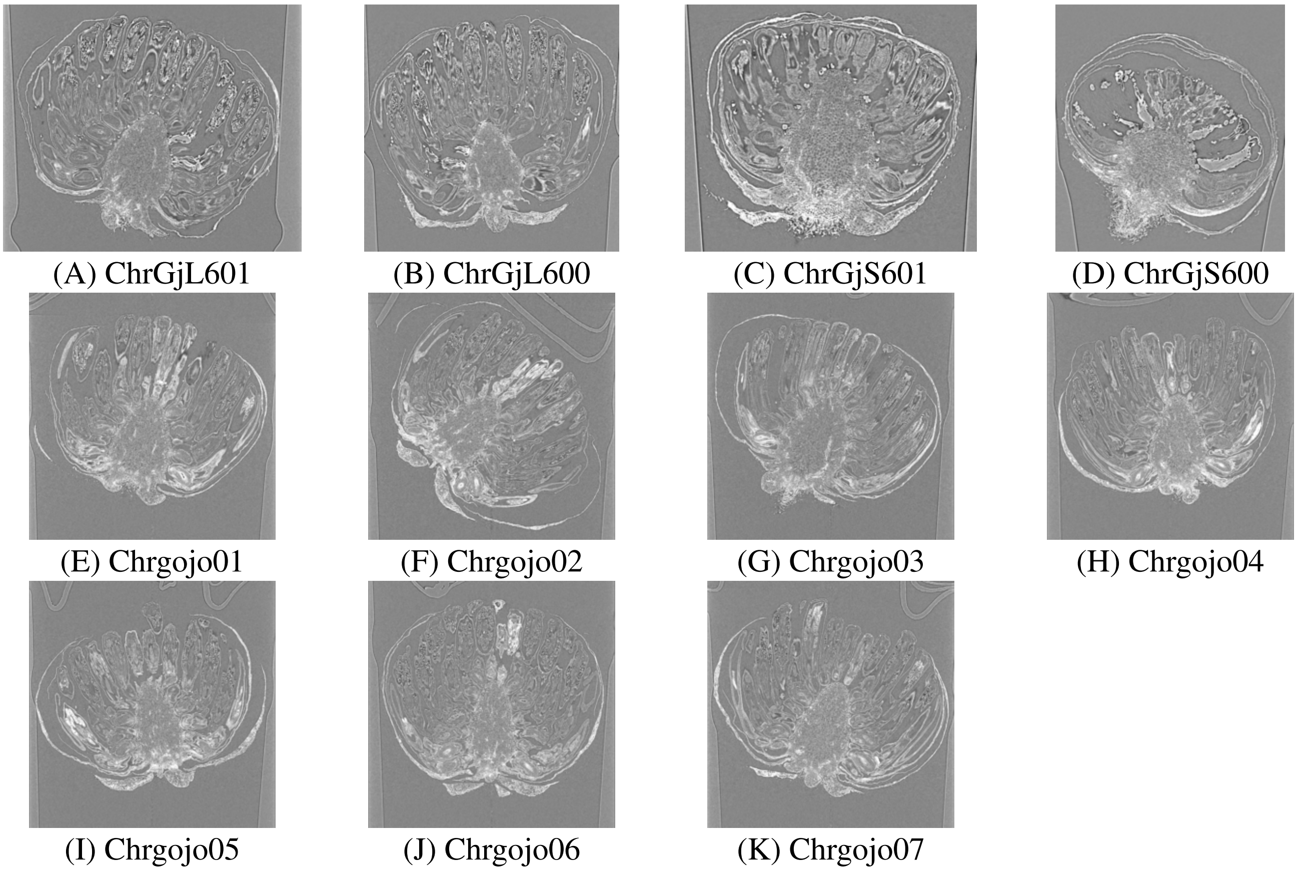

(A) ChrGjL601 (B) ChrGjL600 (C) ChrGjS601 (D) ChrGjS600

(E) Chrgojo01 (F) Chrgojo02 (G) Chrgojo03 (H) Chrgojo04

(I) Chrgojo05 (J) Chrgojo06 (K) Chrgojo07

Figure S1. CT slice images of the *C. seticuspe* samples used in the experiment.

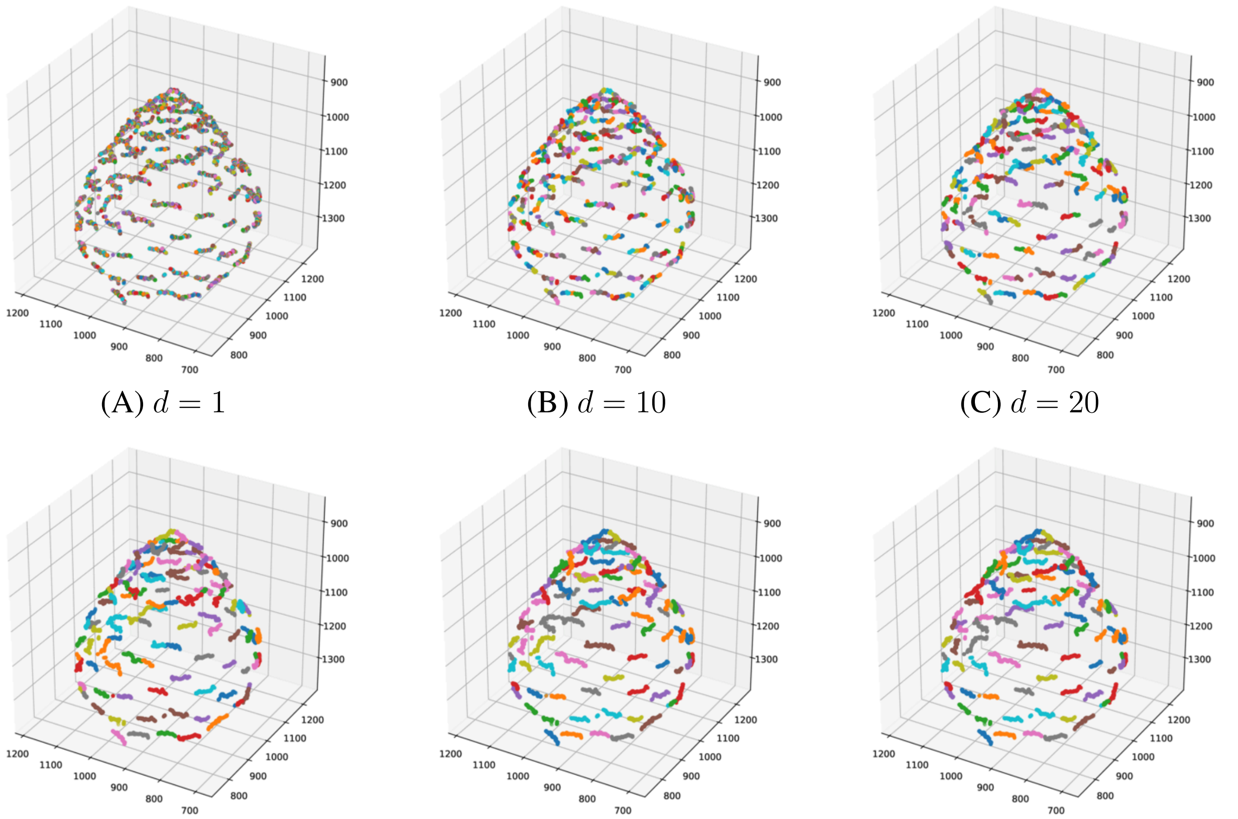

(A) $d = 1$ (B) $d = 10$ (C) $d = 20$

(D) $d = 30$ (E) $d = 40$ (F) $d = 50$

Figure S2. ChrGjL600 clustering result when the training and test data were from the same bud sample. The cluster coloring method is the same as in Figure 6.

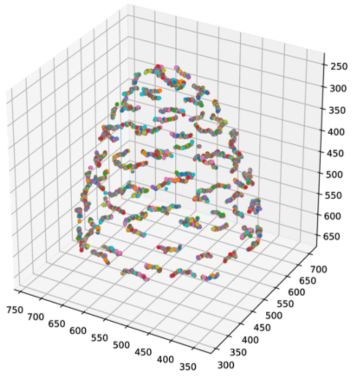

(A) $d = 1$

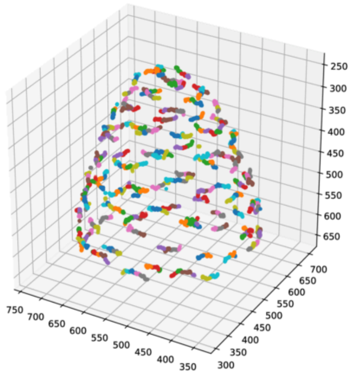

(B) $d = 10$

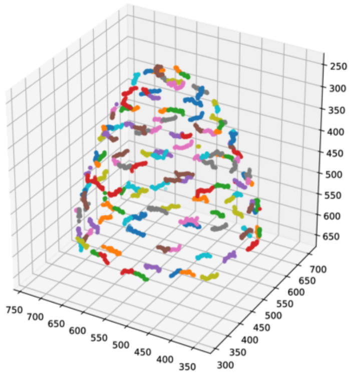

(C) $d = 20$

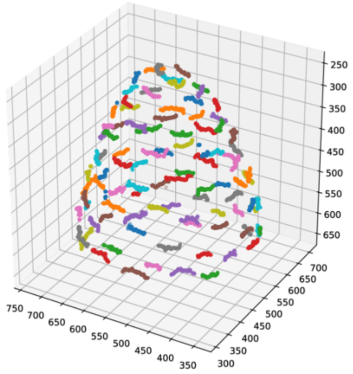

(D) $d = 30$

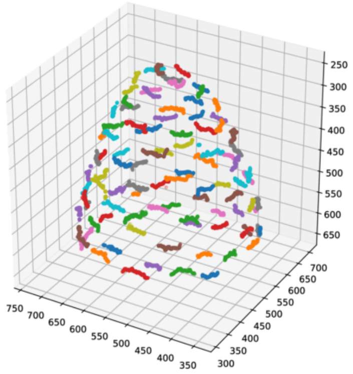

(E) $d = 40$

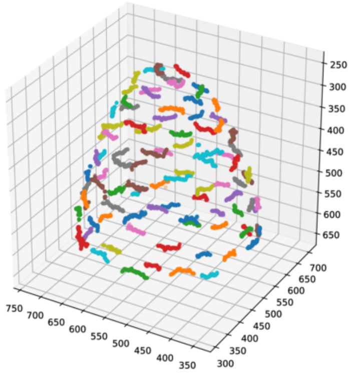

(F) $d = 50$

Figure S3. ChrGjS601 clustering result when the training and test data were from the same bud sample. The cluster coloring method is the same as in Figure 6.

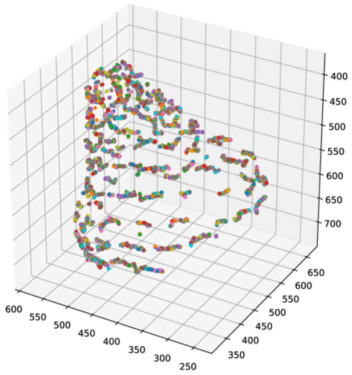

(A) $d = 1$

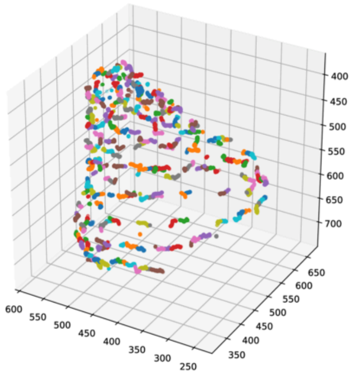

(B) $d = 10$

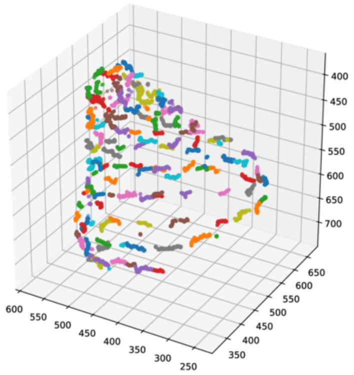

(C) $d = 20$

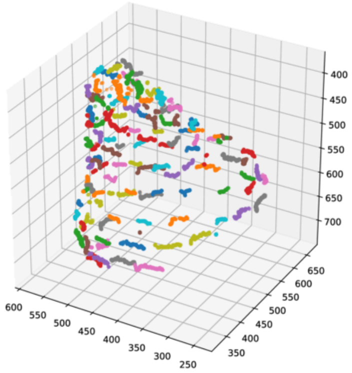

(D) $d = 30$

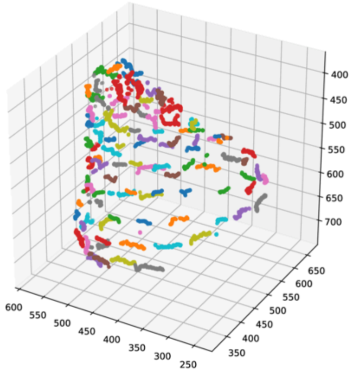

(E) $d = 40$

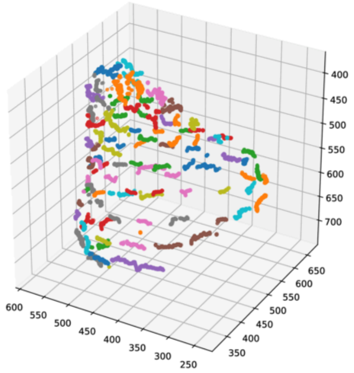

(F) $d = 50$

Figure S4. ChrGjS600 clustering result when the training and test data were from the same bud sample. The cluster coloring method is the same as in Figure 6.

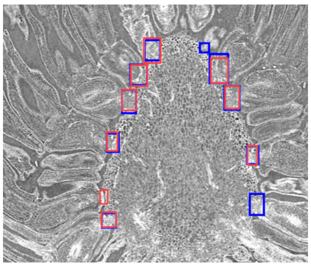

(A) Chrgojo04

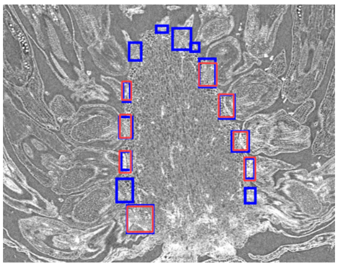

(B) Chrgojo05

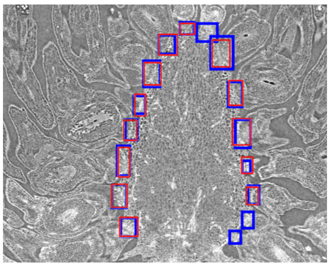

(C) Chrgojo06

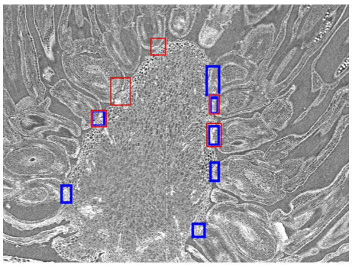

(D) Chrgojo07

Figure S5. Example of detection results for the contact point between the florets and the receptacle for each test bud sample when the training and test samples were different. The blue rectangles show the ground truth of the contact points. The red bounding boxes show the detection results. The independent blue rectangle in (A), (B), (C) and (D) and independent red rectangle in (A) and (D) indicate failure in detection (false negative) and wrong detection (false positive), respectively.

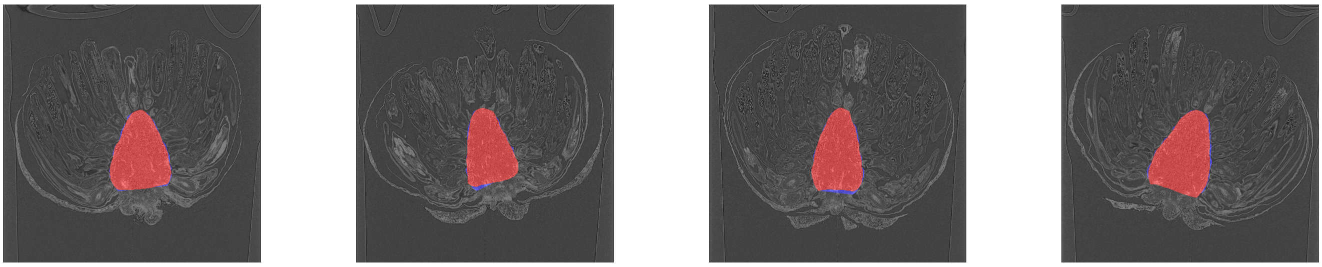

(A) Chrgojo04 (B) Chrgojo05 (C) Chrgojo06 (D) Chrgojo07

Figure S6. Examples of receptacle segmentation results overlay ground truth for each bud sample when the training and test samples were different. The red regions show the segmentation results, and the blue regions show the ground truth that does not overlap.

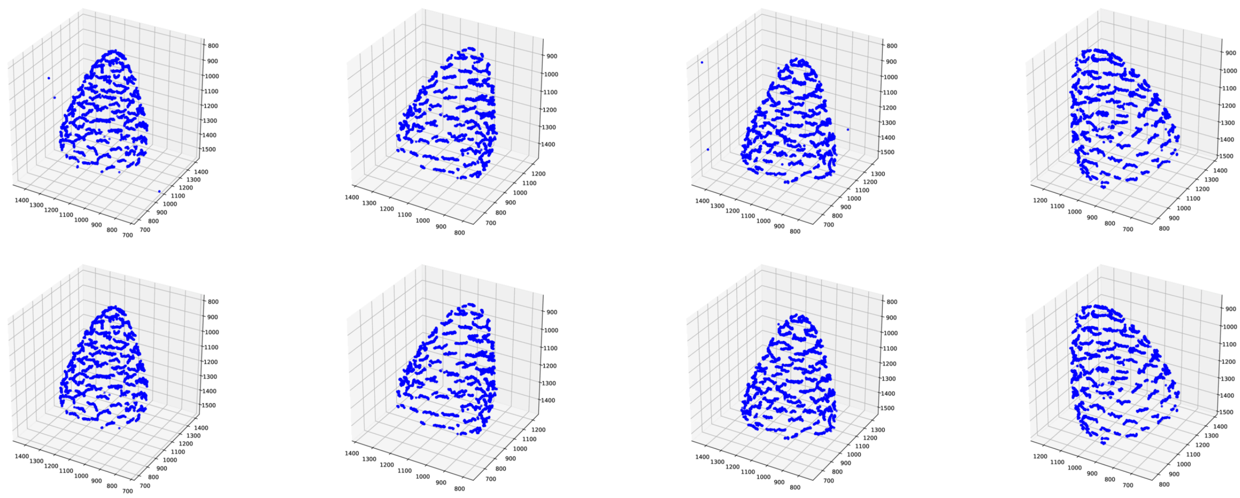

(A) Chrgojo04 (B) Chrgojo05 (C) Chrgojo06 (D) Chrgojo07

Figure S7. 3D integrated contact point detection results for each bud sample when the training and test samples were different. The top and bottom rows show the results before and after removing false positives.

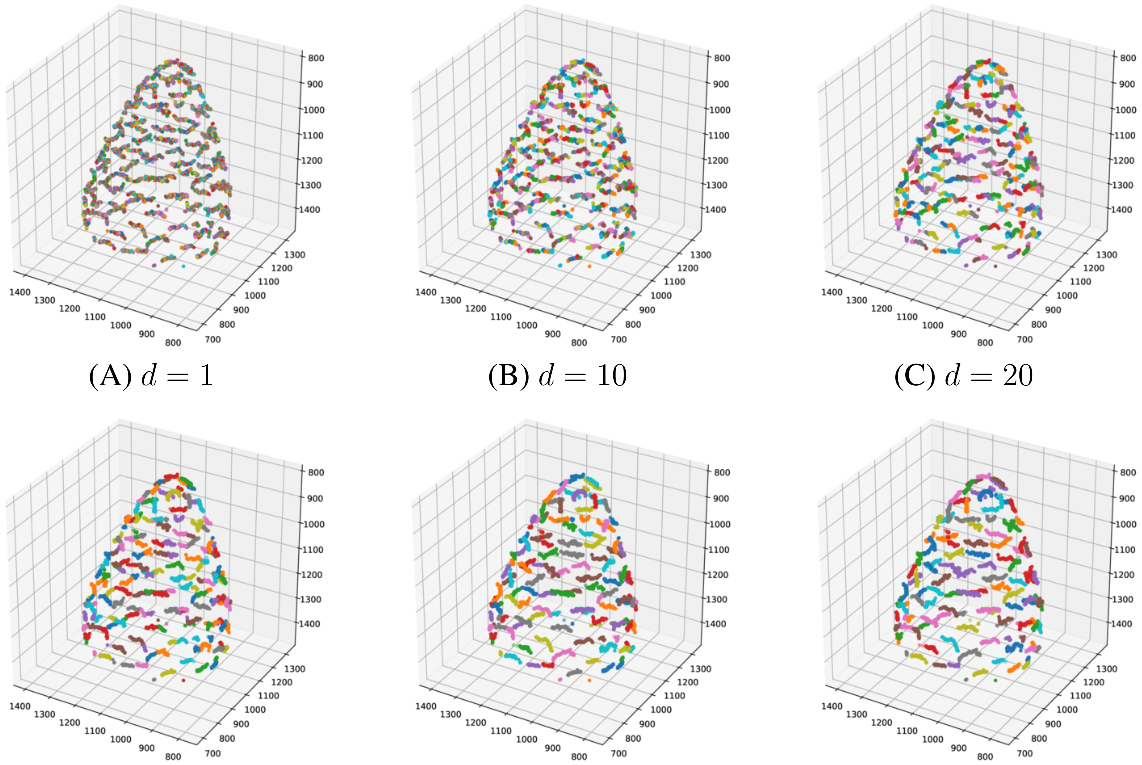

(A) $d = 1$ (B) $d = 10$ (C) $d = 20$

(D) $d = 30$ (E) $d = 40$ (F) $d = 50$

Figure S8. Chrgojo04 clustering result when the training and test data were from the different bud samples. The cluster coloring method is the same as in Figure 6.

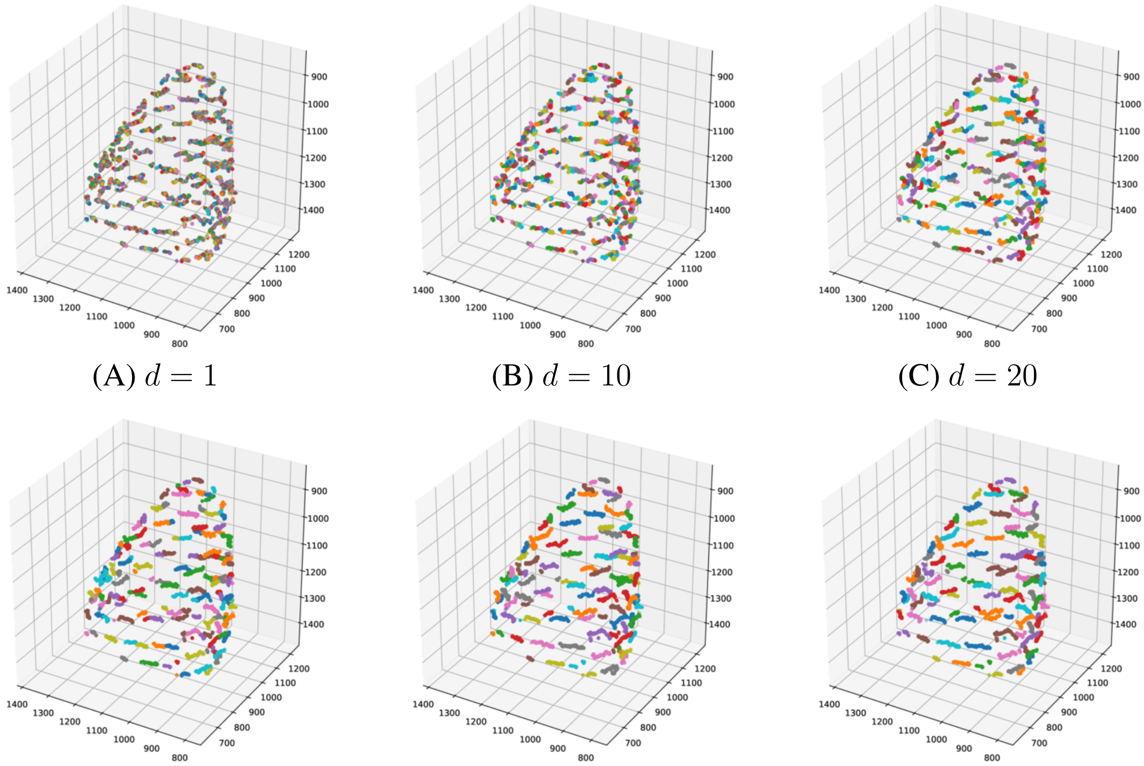

(A) $d = 1$ (B) $d = 10$ (C) $d = 20$

(D) $d = 30$ (E) $d = 40$ (F) $d = 50$

Figure S9. Chrgojo05 clustering result when the training and test data were from the different bud samples. The cluster coloring method is the same as in Figure 6.

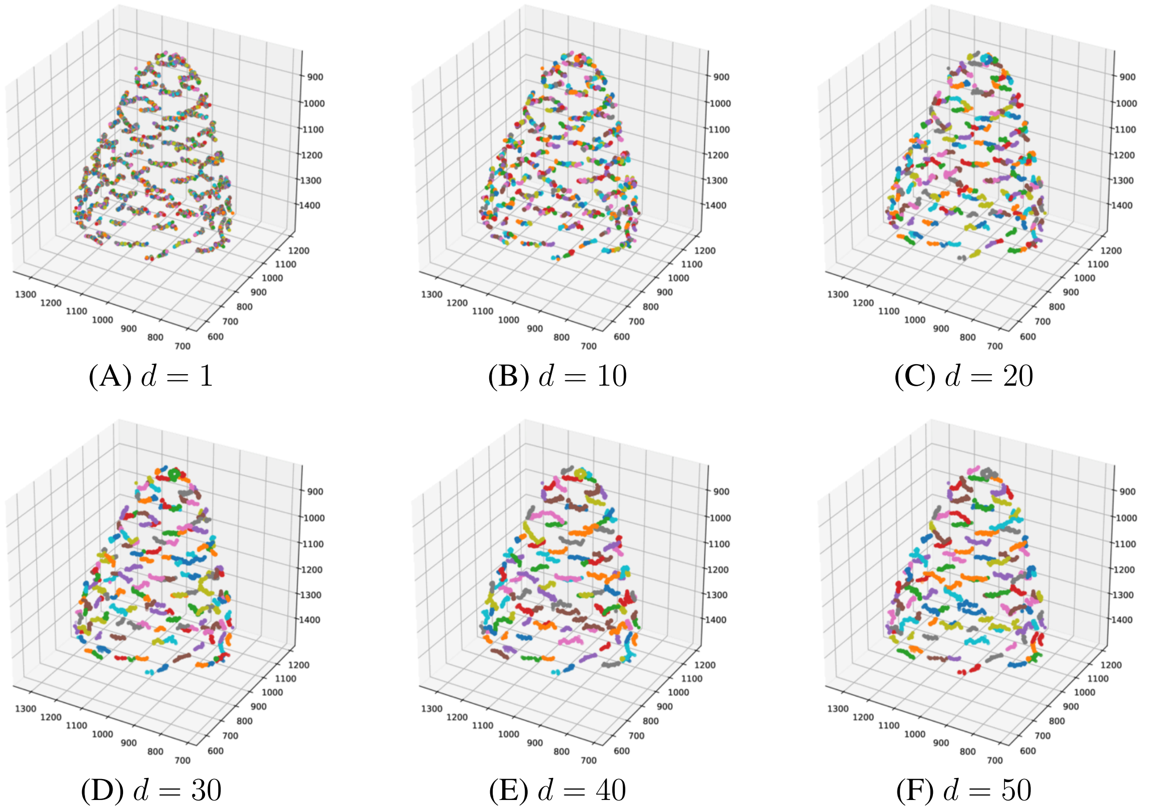

(A) $d = 1$ (B) $d = 10$ (C) $d = 20$

(D) $d = 30$ (E) $d = 40$ (F) $d = 50$

Figure S10. Chrgojo06 clustering result when the training and test data were from the different bud samples. The cluster coloring method is the same as in Figure 6.

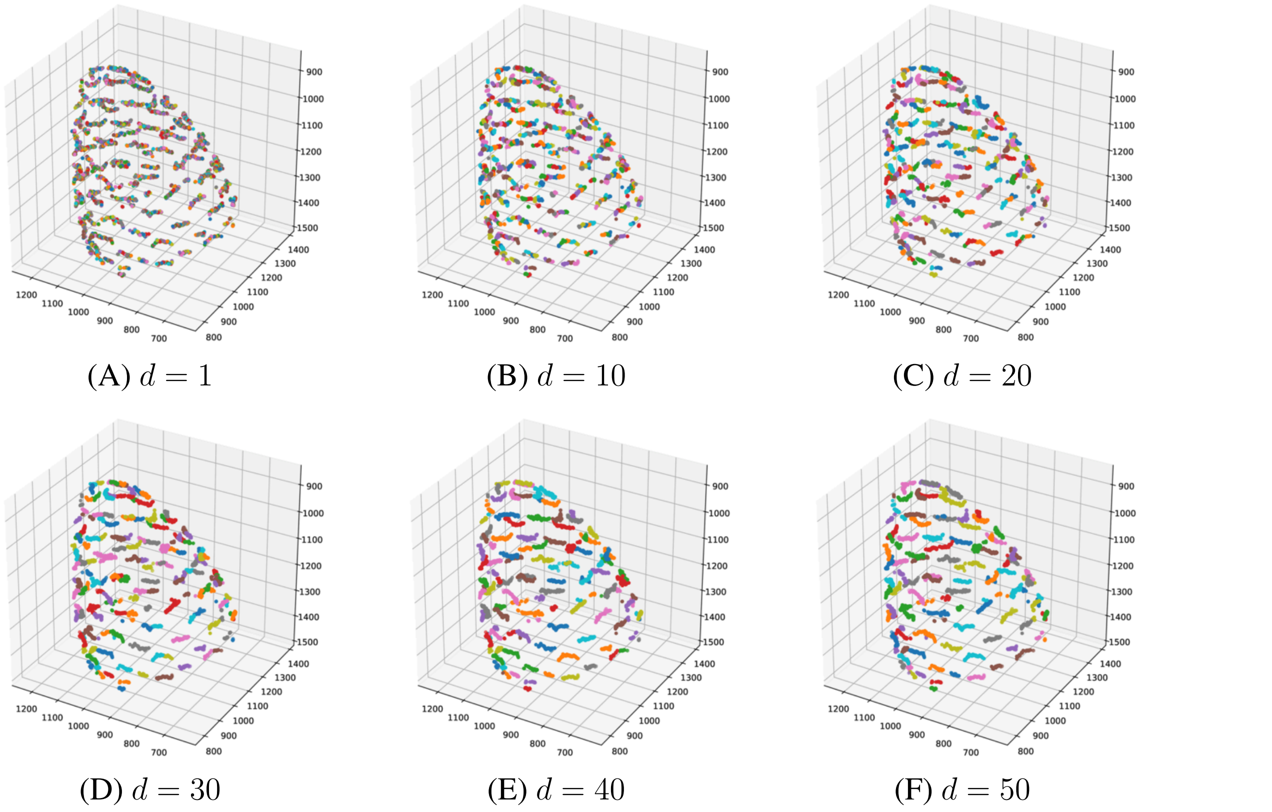

Figure S11. Chrgojo07 clustering result when the training and test data were from the different bud samples. The cluster coloring method is the same as in Figure 6.

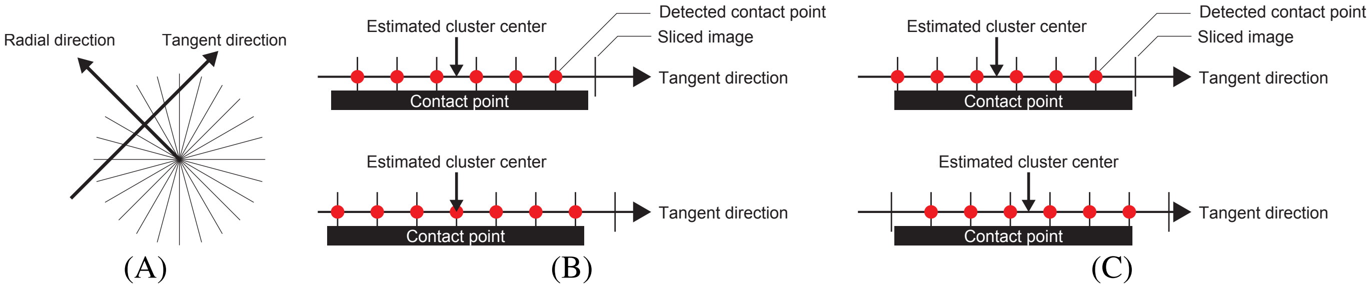

Figure S12. Analysis of potential errors in estimating the center of a contact point. (A) Two directions for analyzing errors. (B) Minimum and (C) maximum error cases in different slicing, respectively.

Table S1. Cropping region for each bud sample.

Sample ID	Crop region (upper left,	lower right)
ChrGjL601	(550, 650),	(1440, 1390)
ChrGjL600	(550, 760),	(1350, 1480)
ChrGjS601	(240, 200),	(820, 730)
ChrGjS600	(180, 330),	(750, 820)
Chrgojo01	(470, 770),	(1520, 1590)
Chrgojo02	(530, 830),	(1580, 1640)
Chrgojo03	(420, 670),	(1610, 1660)
Chrgojo04	(530, 750),	(1490, 1560)
Chrgojo05	(490, 750),	(1560, 1580)
Chrogjo06	(450, 750),	(1470, 1560)
Chrgojo07	(470, 720),	(1600, 1600)

Table S2. AP of contact point detection during training and validation, and test data are from different bud samples.

	Sample ID	AP(0.5:0.95)	AP50	AP75
val	Chrgojo01	0.4746	0.9557	0.3754
	Chrgojo02	0.3993	0.8946	0.3073
	Chrgojo03	0.4671	0.9374	0.3945
test	Chrgojo04	0.5151	0.8992	0.5998
	Chrgojo05	0.5344	0.9442	0.5705
	Chrgojo06	0.5487	0.9647	0.5437
	Chrgojo07	0.2937	0.7957	0.1473

Table S3. Dice coefficient for receptacle segmentation when training, validation, and test data were obtained from different bud samples.

	Sample ID	Dice coefficient mean
val	Chrgojo01	0.9551
	Chrgojo02	0.9577
	Chrgojo03	0.9671
test	Chrgojo04	0.9664
	Chrgojo05	0.9616
	Chrgojo06	0.9662
	Chrgojo07	0.9618
